# Bacterioplankton Biogeography of the Atlantic Ocean: A Case Study of the Distance-Decay Relationship

**DOI:** 10.3389/fmicb.2016.00590

**Published:** 2016-04-26

**Authors:** Mathias Milici, Jürgen Tomasch, Melissa L. Wos-Oxley, Johan Decelle, Ruy Jáuregui, Hui Wang, Zhi-Luo Deng, Iris Plumeier, Helge-Ansgar Giebel, Thomas H. Badewien, Mascha Wurst, Dietmar H. Pieper, Meinhard Simon, Irene Wagner-Döbler

**Affiliations:** ^1^Group Microbial Communication, Helmholtz-Center for Infection ResearchBraunschweig, Germany; ^2^Group Microbial Interactions and Processes, Helmholtz-Center for Infection ResearchBraunschweig, Germany; ^3^UMR 7144 - Sorbonne Universités, UPMC Univ Paris 06, Station Biologique de RoscoffRoscoff, France; ^4^Centre National de la Recherche Scientifique, UMR 7144, Station Biologique de RoscoffRoscoff, France; ^5^Department of Biology of Geological Processes, Institute for Chemistry and Biology of the Marine Environment, University of OldenburgOldenburg, Germany

**Keywords:** bacterioplankton, marine bacteria, microalgae, distance-decay relationship, Particle associated bacteria, biogeography, macroecology, oceanographic province

## Abstract

In order to determine the influence of geographical distance, depth, and Longhurstian province on bacterial community composition and compare it with the composition of photosynthetic micro-eukaryote communities, 382 samples from a depth-resolved latitudinal transect (51°S–47°N) from the epipelagic zone of the Atlantic ocean were analyzed by Illumina amplicon sequencing. In the upper 100 m of the ocean, community similarity decreased toward the equator for 6000 km, but subsequently increased again, reaching similarity values of 40–60% for samples that were separated by ~12,000 km, resulting in a U-shaped distance-decay curve. We conclude that adaptation to local conditions can override the linear distance-decay relationship in the upper epipelagial of the Atlantic Ocean which is apparently not restrained by barriers to dispersal, since the same taxa were shared between the most distant communities. The six Longhurstian provinces covered by the transect were comprised of distinct microbial communities; ~30% of variation in community composition could be explained by province. Bacterial communities belonging to the deeper layer of the epipelagic zone (140–200 m) lacked a distance-decay relationship altogether and showed little provincialism. Interestingly, those biogeographical patterns were consistently found for bacteria from three different size fractions of the plankton with different taxonomic composition, indicating conserved underlying mechanisms. Analysis of the chloroplast 16S rRNA gene sequences revealed that phytoplankton composition was strongly correlated with both free-living and particle associated bacterial community composition (R between 0.51 and 0.62, *p* < 0.002). The data show that biogeographical patterns commonly found in macroecology do not hold for marine bacterioplankton, most likely because dispersal and evolution occur at drastically different rates in bacteria.

## Introduction

Our understanding of marine bacterioplankton diversity and biogeography has been revolutionized by high-throughput sequencing in combination with extensive sampling efforts. Both the depth of sampling (geographical ranges covered) and the depth of community analysis (number of sequences per sample generated) now make the investigation of fundamental concepts of ecology feasible. The distance-decay relationship describes how the similarity in species composition between two or more communities varies with the geographical distance that separates them (Morlon et al., 2008). Usually community similarity decreases with increased geographical distance because of dispersal limitation and local adaptation (Nekola and White, 1999; Soininen et al., 2007a; Nemergut et al., 2013). Evidence for such a relationship in aquatic microbial communities is weak and controversial. A negative distance-similarity relationship was observed in an analysis of 438 samples from globally distributed sites across the oceans, encompassing coastal waters and sediments, surface and deep waters, and deep sea sediments (Zinger et al., 2011, 2014). Nevertheless, the slopes of the bacterial distance-decay curves were much smaller than those observed for plants and animals, and were most strongly affected by the number of samples analyzed and by sequencing depth, underlining the necessity of coupling high-throughput sequencing approaches with wide geographical gradients (Zinger et al., 2014). By contrast, other studies found no distance-decay relationship or only over a restricted geographical range. The *Tara* Oceans expedition provides the largest and most complex metagenomics dataset of the marine bacterioplankton available to date (Sunagawa et al., 2015). It showed a weak decrease in bacterial community similarity with increasing distance between sampling sites, but only up to about 5000 km. At greater distance, similarity did not decrease further and even increased again (Sunagawa et al., 2015). A study of β-diversity of ammonia oxidizing bacteria (AOB) found that distance-decay relationships could be observed on regional scales, but not at a global level when samples from different continents were compared (Martiny et al., 2011). In the South China Sea, a distance-decay relationship was observed for the active component, but not for the total bacterioplankton (Zhang et al., 2014). A study on Ammonia-oxidizing Archaea (AOA) communities along a latitudinal transect in the Atlantic ocean has recently shown that community similarity had a negative linear relationship with distance up to ~6000 km. Afterwards community similarity started to increase again and was highest at the end of the transect (~15,000 km) suggesting a “bipolar” distribution of the AOA communities (Sintes et al., 2015). Thus, evidence is accumulating that bacterioplankton communities might follow a scale dependent distance-decay relationship.

The drivers behind biogeographical patterns are mainly selection and dispersal (Hanson et al., 2012). Ocean currents are a driver for microbial biogeography, as demonstrated by a study of advection in the Southern Ocean (Wilkins et al., 2013). In the deep Arctic Ocean it was shown that water masses characterized by distinct thermohaline properties harbor different microbial communities which are stable over thousands of kilometers (Galand et al., 2010). Clustering of microbial communities according to the water mass has also been observed in the deep Atlantic Ocean (Agogue et al., 2011). In the epipelagic zone of the ocean, the constant mixing of the water column enhances dispersal of marine bacteria, which is achieved by gyre circulation (Walsh et al., 2015) and up-and down-welling processes (Bergen et al., 2015).

However, the ability of bacteria to colonize a niche does not only depend upon their dispersal rate but on selective processes (de Wit and Bouvier, 2006; Hanson et al., 2012), among which temperature and salinity have an important role. While temperature explained 61% of the variance of the *Tara* Oceans bacterioplankton samples; when using only species abundance as information, temperature could be predicted with an explained variance of 86% (Sunagawa et al., 2015). Salinity was shown to select for different microbial communities along a gradient in the Baltic Sea, and in this study it could also be shown that brackish conditions were not characterized by reduced diversity as observed for macro-organisms (Herlemann et al., 2011).

Selection can not only be executed by environmental parameters, but also and perhaps more importantly by biotic interactions. The phycosphere defined as a layer surrounding photosynthetic micro-eukaryotes that extends outwards from the algal cell as well as the surface of microalgae represent unique environments that are hotspots of interactions (Gast et al., 2009; Amin et al., 2012). Thus, marine micro-algae can also be expected to significantly influence the composition of bacterioplankton communities. Several studies described changes in bacterioplankton communities during algal blooms (Teeling et al., 2012; Wemheuer et al., 2014; Yang et al., 2015), but no systematic evaluation of the influence of photosynthetic micro-eukaryotic diversity on bacterioplankton communities has been conducted so far on a broad geographical scale.

Phytoplankton growth is the driver of marine productivity, and so the concept of biogeochemical provinces based on the dynamics of surface temperature, salinity, bathymetry, and chlorophyll *a* concentration was developed to predict algal blooms in the ocean (Longhurst et al., 1995; Longhurst, 1998; Reygondeau et al., 2013). Thus, these provinces provide an ecologically useful framework for interpreting biogeographical data. While the role of Longhurstian provinces for pico-phytoplankton is well documented (Li and Harrison, 2001), their influence on bacterial biogeography is unclear. Previous studies on the Atlantic Ocean have suggested that province-related bacterial signatures exist (Friedline et al., 2012) while others have demonstrated evidence for ammonia oxidizing archaea signatures (Sintes et al., 2015).

To this end, here we analyzed a spatially highly resolved set of water samples from the epipelagic zone of the Atlantic Ocean covering a distance of ~12,000 km to investigate the following questions:

1. On which geographical scale and for which depth layer of the epipelagic zone (between 20 and 200 m) is a distance-decay relationship for bacterioplankton observed, if any?
2. Do Longhurstian provinces harbor specific microbial communities?
3. Is there a correlation between the composition of photosynthetic micro-eukaryotic communities and bacterial communities over a large geographical range?

Community composition of bacteria was determined by Illumina sequencing of 16S rRNA gene amplicons, and plastid rRNA gene amplicons were used for an assessment of community composition of photosynthetic eukaryotes. The taxonomic composition of the communities and co-occurrence networks are described in a related manuscript (Milici et al., 2016a).

Water samples were fractionated by sequential filtration into three size classes. It has been suggested that the size of the organisms could influence the distance-decay relationship (and other biogeographical patterns) and this has been demonstrated for micro-eukaryotes (Hillebrand, 2004; Soininen et al., 2007b, 2013; Astorga et al., 2012; Soininen, 2012, 2014). Therefore, we tested all biogeographical patterns for free-living bacteria compared to those that were attached to small (between 3 and 8 μm) and large particles (>8 μm).

## Materials and methods

### Sampling

**Figure 1:**
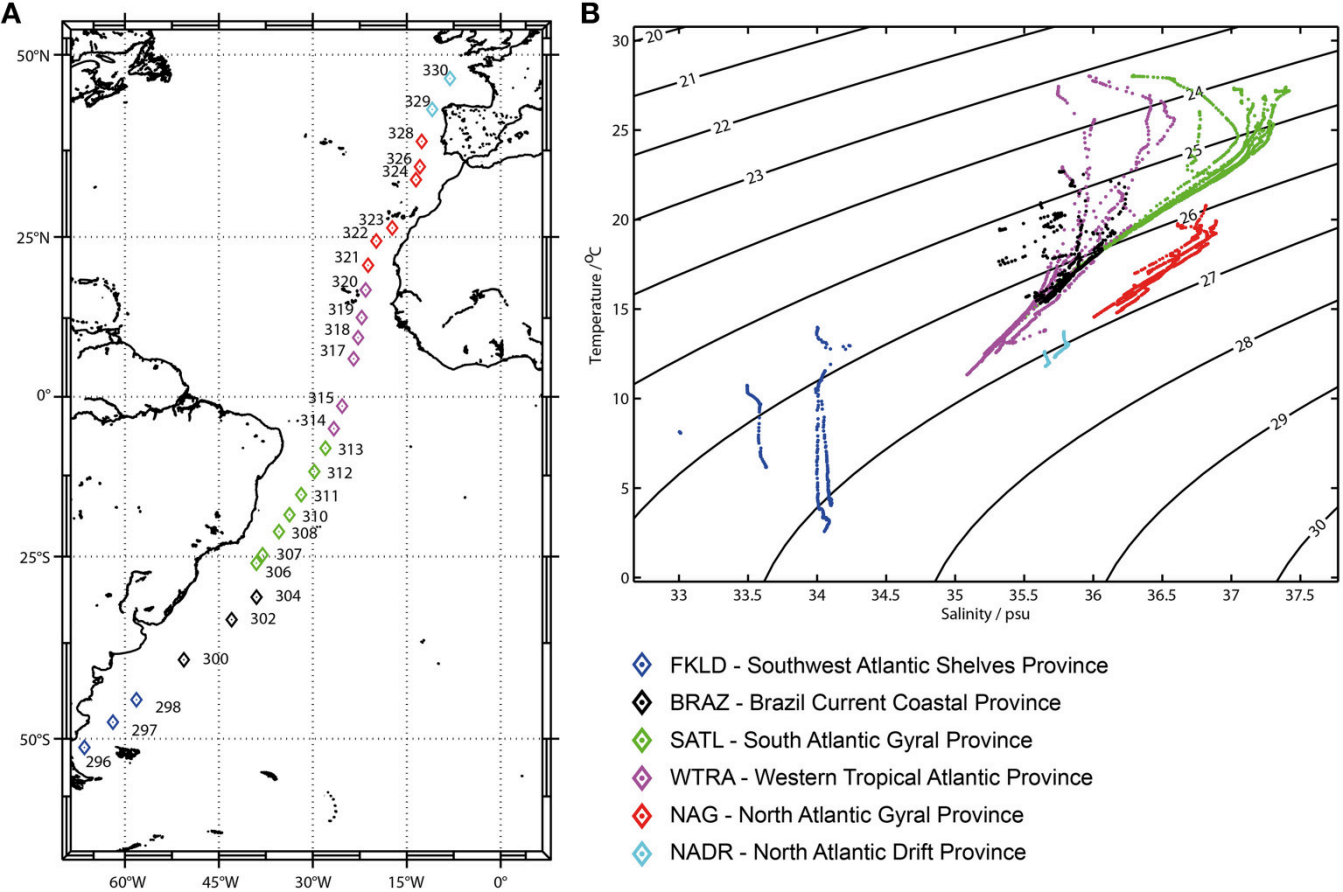
**Sampling sites and oceanographic provinces: (A) Sampling sites**. Color codes display the six oceanographic provinces identified in the temperature-salinity (TS) diagram **(B)**. The six oceanographic provinces were defined on the basis of similar properties (temperature and salinity) of the first 200 m of the water column.

Samples were collected during cruise ANT-28/5 (10 April–15 May 2012 with RV Polarstern) at 27 stations across a latitudinal transect in the Atlantic Ocean (51°S–47°N) (Figure 1A). At all stations, samples were consistently collected from five depths of the epipelagic zone: 20, 40, 60, 100, and 200 m; seven samples were taken ±10 m from the designated depths and an additional eight samples were taken from intermediate depths (Table S1). To be able to analyze those samples together with the others, all samples were grouped according to five depth layers: 20, 40, 50–80, 85–120, and 140–200 m (Table S1). Samples were size fractionated via serial filtration as described (Milici et al., 2016b) resulting in three bacterial communities: FL (free-living) for bacteria collected on the 0.22 μm membranes, SPA (small particle associated) for bacteria collected on the 3 μm membranes and LPA (large particle associated) for bacteria collected on the 8 μm membranes. Sampling, serial filtration, DNA extraction, Illumina sequencing, and bioinformatics analyses were performed as described (Milici et al., 2016b). The raw data were submitted to the ENA database (European Nucleotide Archive) and were assigned the BioProject ID: PRJEB11493. The obtained sequences (Table S1) were taxonomically classified. With the reference database SILVA (119 NR) (Pruesse et al., 2007). The OTUs were aligned and classified against a maximum of 100 sequences that had a minimum of 97% similarity with the query sequence, using the lowest common ancestor method (LCA). All OTUs that could not be affiliated unambiguously to the domain “Bacteria” were removed from the dataset (Table S1). Sequences that were classified as “chloroplast” were collected in a separate dataset (Table S1) and analyzed with the PhytoREFdatabase (Decelle et al., 2015) with BLAST version 2.2.28 (Camacho et al., 2009). All reads that had a similarity below 95% to known 16S sequences were excluded from the dataset. Chloroplast sequences retrieved from the 3 and 8 μm membranes were named SP (small phytoplankton) and LP (large phytoplankton), respectively.

### Measurements of chlorophyll *a*

*In situ* chlorophyll *a* (Table S1) concentrations were determined on board in the dark by using a Turner fluorometer for lower values according to the methods described in Edler (1979) and Evans (1987). 1.0–4.0 L of seawater were filtered through Whatman GF/F glassfiber filters (25 mm diameter) and stored at −20°C until a subsequent 90%-acetone filter extraction (2 h at −20°C). Calibration of the fluorometer was carried out with a standard chlorophyll solution (Sigma).

### Statistical analysis

Statistical analyses were performed with PRIMER [v.6.1.6, PRIMER-E, Plymouth Marine Laboratory, Plymouth, UK; (Clarke and Gorley, 2006)], with the add-on PERMANOVA+ (v. 1.0.6 PRIMER-E, Plymouth Marine Laboratory, Plymouth, UK; Anderson et al., 2006) and the statistical program R (http://www.Rproject.org/, v. 3.0.1) with the library vegan: Community Ecology Package (v. 2.0–8). The number of sequence reads per OTU was normalized to the total number of sequence reads per sample. (i.e., the absolute abundances were transformed in relative abundances expressed in %). The generated table was employed in all further analyses. To investigate beta diversity, Bray-Curtis similarity was calculated and the matrix generated was explored with principal coordinates analysis (PCO). To assess the effect of environmental parameters vector overlay was performed based on Pearson correlation with an *R*-value higher than 0.4 with the first two axes of the PCO. Correlation of the similarity matrices with algal distribution (plastidial 16S) was performed using a non-parametric mantel-type test (RELATE) with Spearman-rank correlation (999 permutations). Distance-based multivariate analysis for a linear model (DistLM) was carried out with forward selection and adjusted *r*^2^ selection criterion (999 permutations) to calculate the variation explained by environmental parameters and geographical position. Permutational analysis of multivariate variance (PERMANOVA) with 999 permutations was used to test the effect of the factors province and depth layers in a 2-way crossed design separately for each size fractionated community. The ANOSIM test was used to assess significant differences among communities from different filter sizes with 999 permutations.

## Results

### Province characterization and hydrography

Province definition was based on the oceanographic data from the 27 sampled stations (Figure 1A). Temperature and salinity profiles of the epipelagic zone (20–200 m) were used to generate T-S diagrams. Six oceanographic provinces could be identified (Figure 1B) and were named according to the classification of Longhurst (Longhurst, 1998): Southwest Atlantic Shelves Province (FKLD) 51°S–45°S, Brazil Current Coastal Province (BRAZ) 40°S–30°S, South Atlantic Gyral Province, (SATL) 26°S–5°S, Western Tropical Atlantic Province (WTRA) 2°S–17°N, North Atlantic Gyral Province (NAG) 20°N–38°N, and North Atlantic Drift Province (NADR) 42°N–47°N. These provinces do not have exactly the same boundaries as those described by Longhurst, since allocation was based on the environmental data collected during the oceanographic cruise. From the T-S diagram in Figure 1B, it clearly emerges that the FKLD province harbors different thermohaline properties compared to the other provinces along the transect, where this area had the lowest salinity and temperatures, while the other five provinces were more similar to each other although distinguishable according to their temperature- salinity profiles. The thermohaline properties of the six oceanographic provinces are provided in Tables S2,S3.

### Biogeographical patterns of bacterioplankton community composition

**Figure 2:**
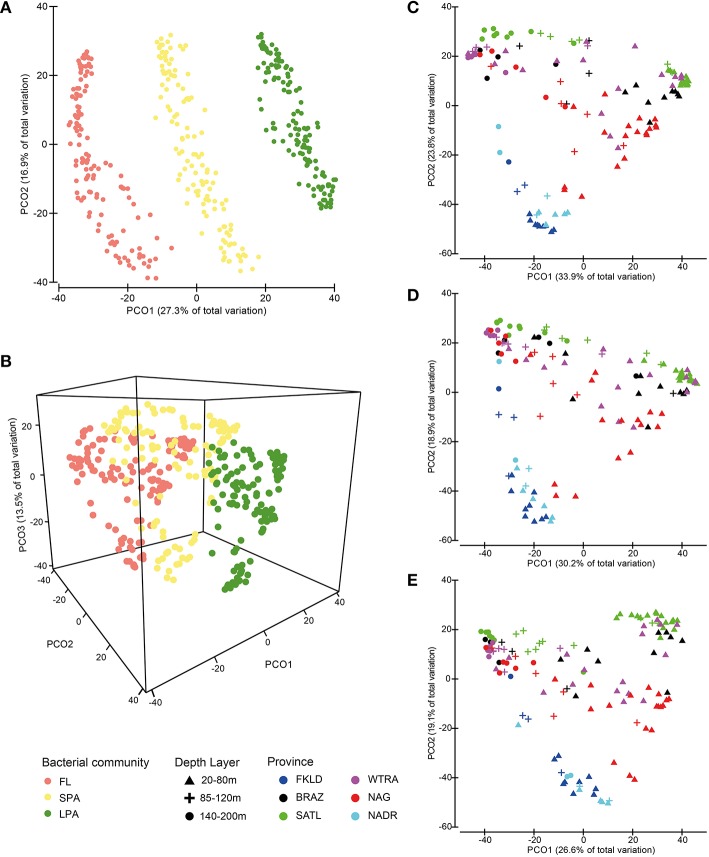
**Similarities between bacterial communities**. Bray-Curtis similarity was calculated for standardized abundance data at the OTU level. Panels **(A**,**B)** show similarities between the three size fractions of the bacterioplankton FL (free-living), SPA (small particle associated) and LPA (large particle associated). Panels **(C,D,E)** show similarities for each of the three communities separately. Bacterial abundances were standardized by the total for **(A,B,C,D,E)** and root transformed for **(A,B)**. Color codes discriminate the size fractions of the communities for **(A,B)**, while they distinguish provinces for **(C,D,E)**. Depth layers are shown by different symbols.

The three bacterioplankton communities FL (free living), SPA (small particle associated), and LPA (large particle associated) were significantly different (ANOSIM test *P* < 0.001, global *R* = 0.72). PCO (Figures 2A,B) displayed separation of the three communities that were discriminated along the PCO1 according to the filter size from left to right: FL-SPA-LPA. Despite enourmous differences in the composition of the bacterial communities from the three size classes of the plankton (Milici et al., 2016a), PCO showed a conserved pattern for free-living and particle associated bacteria (Figures 2C,D,E), where a clear triangular pattern with three distinguishable groups can be observed. The two principal coordinates separated the samples into three groups, mainly according to depth layer (PCO1) and province (PCO2): a first group positioned at the bottom of the PCO plots comprising samples from the ends of the transect, a second group aligned on the left side of the plots comprising the deep stations (140–200 m) and a third group located to the right side of the plots encompassing the shallow samples (20–80 m) with the 85–120 m samples lying between them. The first group encompassed the samples from the marginal provinces, FKLD and NADR, and the shallow samples (20–85 m) from the last station of the NAG province (station 328). Samples belonging to these provinces showed a low intra-group variation and were not scattered across the PCO1, although they were separated by a huge geographical distance of ~12,000 km. The other two groups of samples included the other provinces, such as BRAZ, SATL, WTRA, and NAG, which showed a high intra-group variation that was due to the bathymetrical distribution along the PCO1, with the vast majority of samples belonging to the deepest layer plotted on the left side of the plots. The communities belonging to the deepest layer (140–200 m) were more homogeneous, while the shallow communities were highly diverse. In fact, the communities from the upper layers exhibited a separation by provinces along the PCO2, with a first subgroup of samples comprising the SATL province and part of the BRAZ and WTRA provinces. A second subset of shallow samples incorporated samples belonging to the NAG province and the remaining samples from the WTRA and BRAZ provinces. The separation according to province was particularly evident for the upper 80 m of the LPA community where the communities were clearly separated according to the province of their origin. The deeper communities were more stable independently from their oceanographic province. Altogether, a conserved biogeographical pattern was observed for Atlantic bacterioplankton communities recovered from three different size fractions of the plankton, independent from the phylogenetic composition of the microbial communities themselves.

### Distance decay of bacterial community similarity in the Atlantic Ocean from 51°S to 47°N

**Figure 3:**
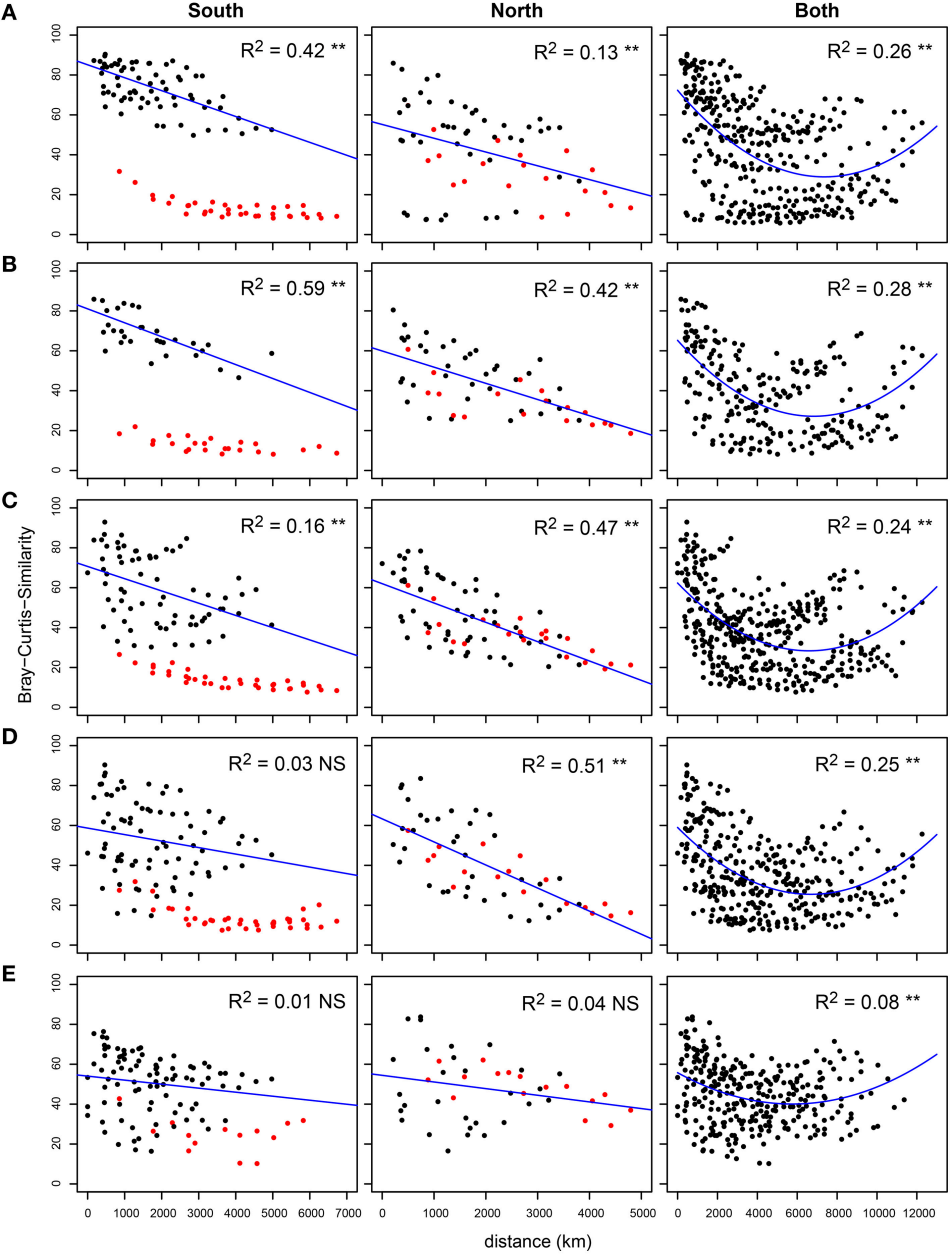
**Distance-decay relationship for free living (FL) communities along the water column**. Bray-Curtis similarity was calculated on standardized abundance data, and plotted against the geographical distance expressed in Km. The transect was analyzed entirely and for the Southern and Northern hemisphere separately, which are displayed from left to right. Samples were divided into five depth layers: **(A)** 20 m, 40 m **(B)**, 50–80 m **(C)**, 85–120 m **(D)**, and 140–200 m **(E)**. Adjusted *R*^2^ value are displayed for each of the linear regressions and second order polynoms on the chart area, as well as the significance level (^**^p < 0.01 and ns *p* > 0.05). For both hemispheres the marginal provinces FKLD and NADR are marked in red. For the Southern hemisphere, due to high variation in community similarity driven by the FKLD province, this province was left out of the calculation for the linear regression.

Both the Southern and the Northern hemisphere of the Atlantic Ocean showed a clear linear distance decay of similarity for bacterial communities in all three size fractions of the plankton (Figure S1). However, when the complete transect was investigated, no linear decrease of similarity was observed; although after roughly 6000 km the similarity increased again and reached values of 40–60% Bray-Curtis similarity when the distance was the largest (~12,000 km). Due to the weak concave structure of the data we could significantly (*P* < 0.001) fit a second order polynomial model, although with small *R*^2^ values (FL: 0.13, SPA: 0.14, LPA: 0.15) most likely because of the effect of depth stratification. Therefore, the five depth layers were analyzed separately (Figure 3 and Figures S2,S3). Our analysis revealed a strong bathymetric pattern for both hemispheres as well as for the whole transect. In all three fractions of the plankton we found that the highest *R*^2^ values were in the upper 120 m of the water column and strongly reduced (and mostly not significant) in the 140–200 m. These results suggest that bacterioplankton communities in the epipelagic zone are strongly stratified, and that different parts of the water column follow different biogeographical patterns. The deepest layer of the epipelagic zone lacked a distance-decay relationship altogether.

### Provincialism and depth stratification of bacterioplankton communities

To determine the effect of province and depth layer on community diversity, the 2-way (crossed) PERMANOVA test (Tables S4–S6) was conducted. It showed a highly significant (*p* < 0.001) and strong provincialism, that explained roughly 30% of the total variation of community composition for bacteria from all three size fractions of the plankton (adjusted *R*^2^ value between 0.32 and 0.30). Depth stratification had a smaller effect when compared to province, accounting for between 16 and 23% of the total variation (*R*^2^ values between 0.23 and 0.16) which was still highly significant (*p* < 0.001). Moreover, a significant (*p* < 0.001) statistical interaction was found between those two factors, indicating that within province there is an effect of depth on bacterial communities and vice versa. This effect accounted for 15–17% of the total variation (adjusted *R*^2^ values between 0.15 and 0.17).

**Figure 4:**
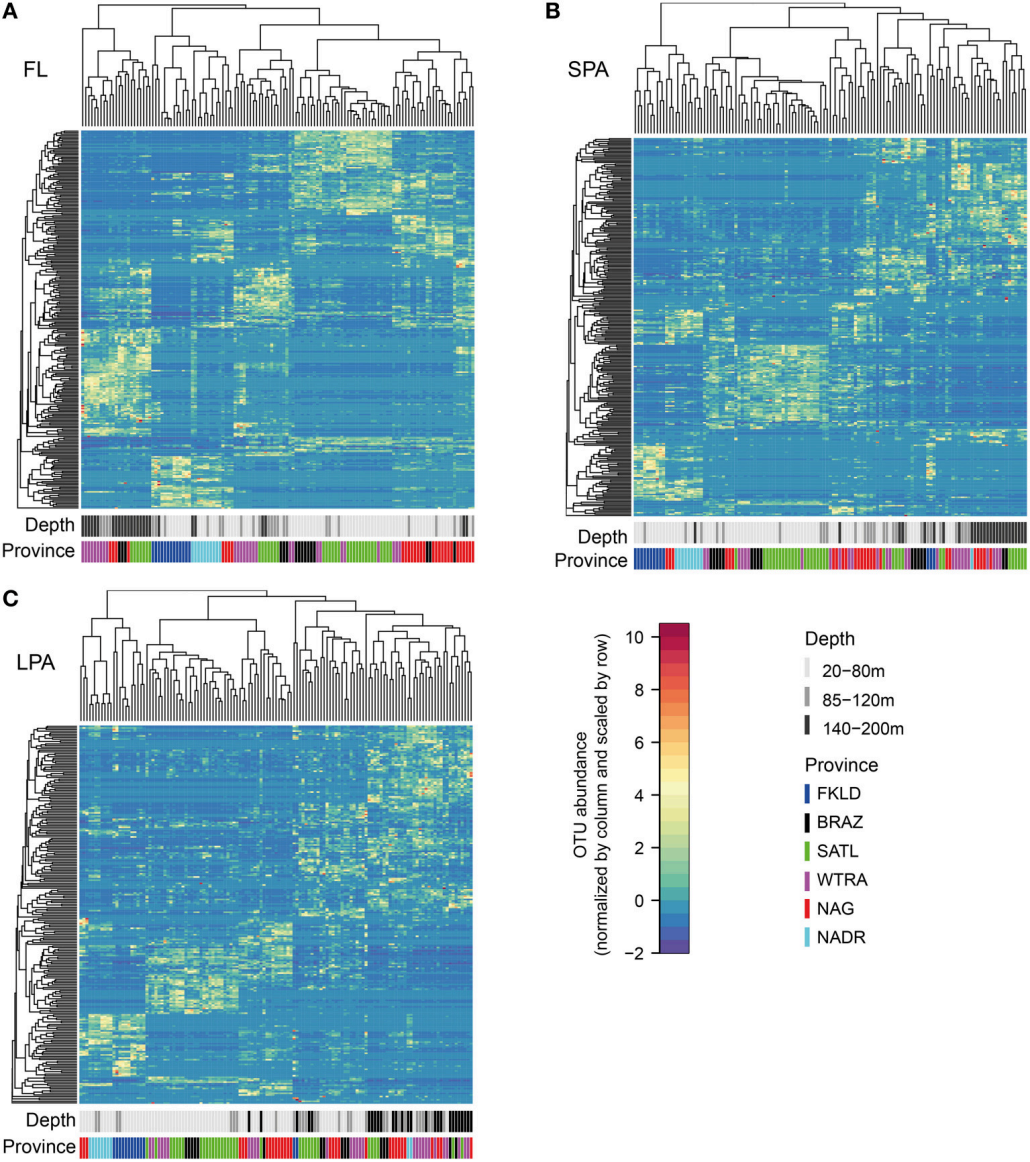
**Co-occurrence analysis of OTUs across provinces and along depth**. Sample size was resampled to obtain an equal number of reads in each sample. Bray-Curtis similarity was calculated and used for the clustering of samples. For a better visualization of the relative abundances the data were scaled by row with values ranging from −2 to 10. The color reflects province and depth layer to visualize clustering of samples. The analysis was performed separately for each of the three size fractions of the plankton FL **(A)**, SPA **(B)**, and LPA **(C)**. On the upper part of the heatmaps sample clustering is reported, while on the left side of the heatmaps the OTU clustering is shown. Every horizontal line therefore indicates an OTU, while every vertical line represents a sample.

**Figure 5:**
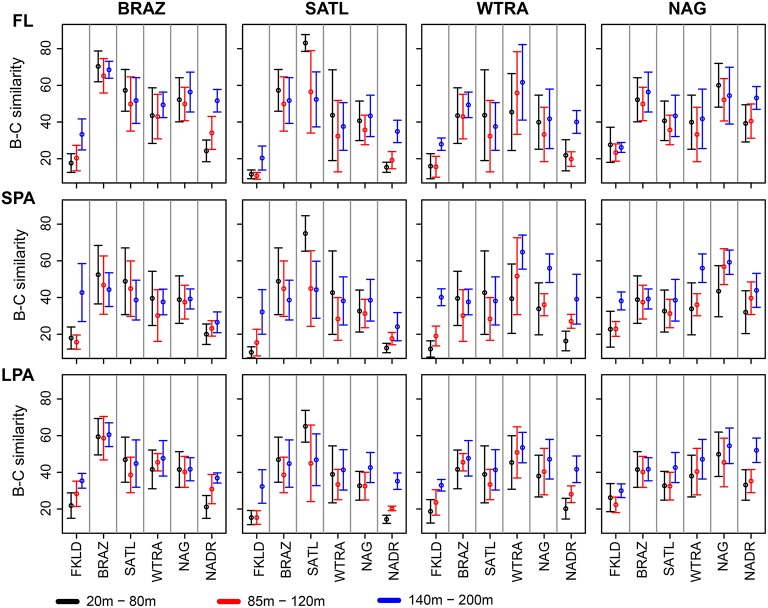
**Province similarity along the water column**. Bray-Curtis similarity was calculated on standardized abundances data. The data were combined into three depth layers: 20–80, 85–120, and 140–200 m for the four intermediate provinces: BRAZ, SATL, WTRA, and NAG. The two marginal provinces FKLD and NADR are showed separately (Figure S4) because of the small number of samples for the 140–200 m depth layer. The average of the Bray-Curtis similarity was calculated for each depth layer of the provinces, and compared against all six provinces. From left to right: BRAZ, SATL, WTRA, and NAG are displayed while from top to bottom the three size fractions of the plankton are shown. The color key shows different depth layers.

Co-occurrence analysis (Figure 4) showed that the patterns described above were due to similar relative abundances of the same sets of OTUs. Sample clustering was guided by the increase or depletion of groups of OTUs that showed a preference for specific provinces and depth layers. This indicates that the vast majority of the OTUs were not ubiquitously distributed along the transect but specialists for certain ecological niches. To demonstrate the combined effect of depth and province, we calculated Bray-Curtis similarity of community composition from each province compared to all other provinces and divided the data into three depth layers (20–80, 85–120, 140–200 m) for the four central provinces (Figure 5) and into two depth layers for the marginal provinces (20–80 and 85–120 m) (Figure S4). The data show that samples within each province were most similar to each other, indicating that oceanographic provinces reflect differing bacterioplankton communities and hence provinces represent a powerful framework in microbial ecology. Moreover, the similarity between different provinces increased with depth layer, i.e., in the lower part of the photic zone similarity was always higher than in the upper part. Finally we found that the FKLD and NADR were the most similar provinces as also suggested by the distance-decay analysis.

### Effect of environmental parameters and geographical location on community structure

Multivariate multiple regression analysis (DistLM) was performed to quantify the variation of bacterioplankton community composition which could be explained by the parameters depth, temperature, salinity, and geographical location for the complete transect (Tables S7–S9). Among the environmental parameters, temperature was the main explanatory variable. Over the complete transect, it accounted for between 17.4% (LPA) and 25% (FL) of the total variability. Its contribution was larger in the Southern than in the Northern hemisphere and it decreased with increasing plankton size (from FL to LPA). Interestingly, temperature explained 33% of the total variation in the FL community of the Southern hemisphere, but only 3% in the LPA community of the Northern hemisphere. The salinity was usually added as the last parameter to the models with a very small contribution (1–2%) of the total variation explained (Tables S7–S9). It was a more important factor in the Northern hemisphere where it contributed 3–9% in the FL and 9% in the LPA community. Latitude and longitude explained comparable amounts of the total variation (7–9%), however its contribution was higher in the Northen hemisphere (17–20%) than in the Southern hemisphere (4–5%).

### Photosynthetic micro-eukaryotes biogeography mirrored bacterioplankton biogeography

**Figure 6:**
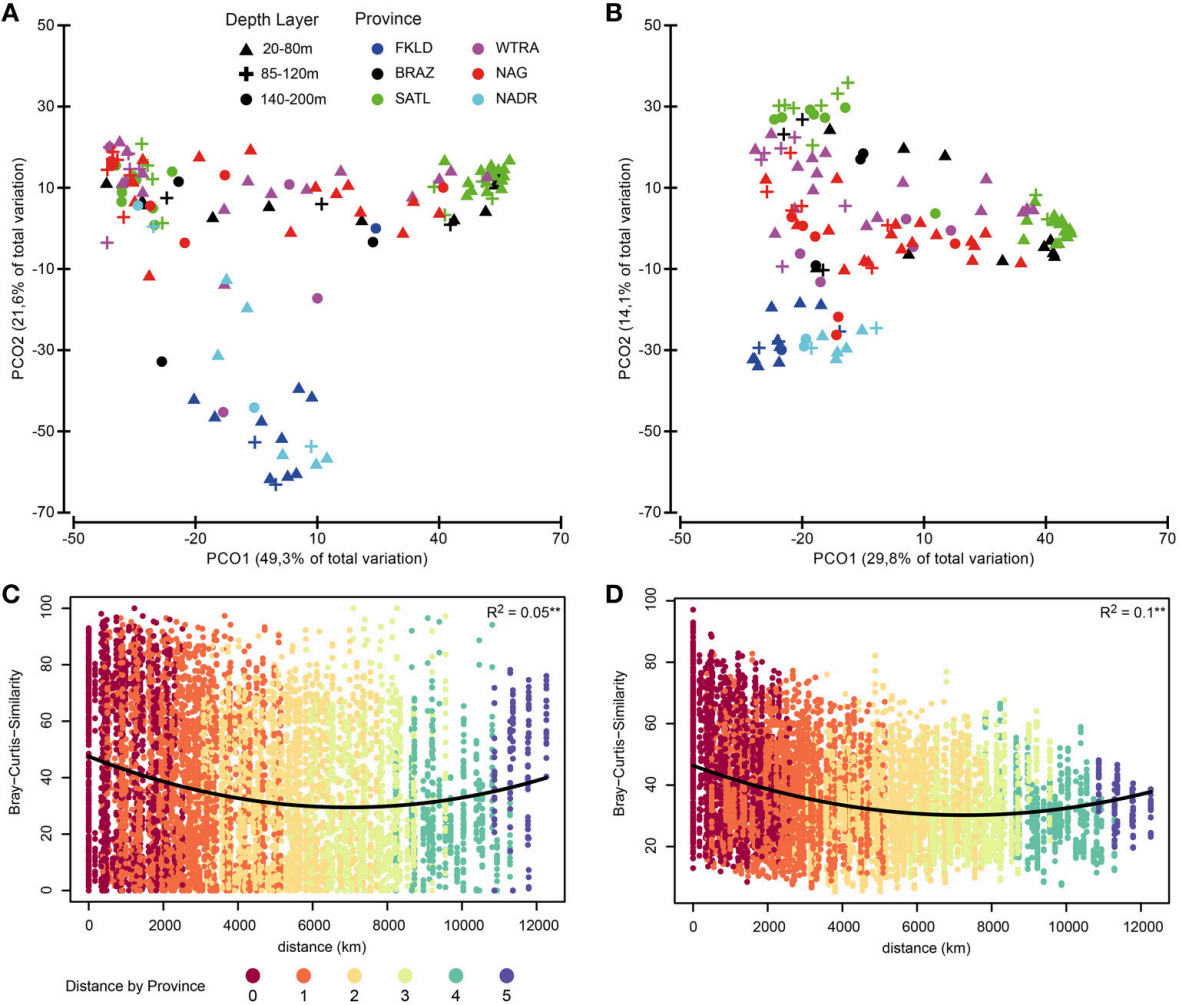
**Biogeography of photosynthetic micro-eukaryotes**. Biogeographical patterns were investigated for the small subunit of RNA sequences from chloroplasts. Bray-Curtis similarity was calculated for standardized abundance data at the OTU level for the SP (small particles) community (left panel, **A**) and the LP (large particles) community (right panel, **B**). Color code discriminate the six oceanographic provinces, while depth layers are shown with different symbols. In panels **(C,D)** the distance-decay relationship for SP and LP communities is displayed. For those two panels the color key indicates the distance between samples (pairwise) express of oceanographic provinces.

**Figure 7:**
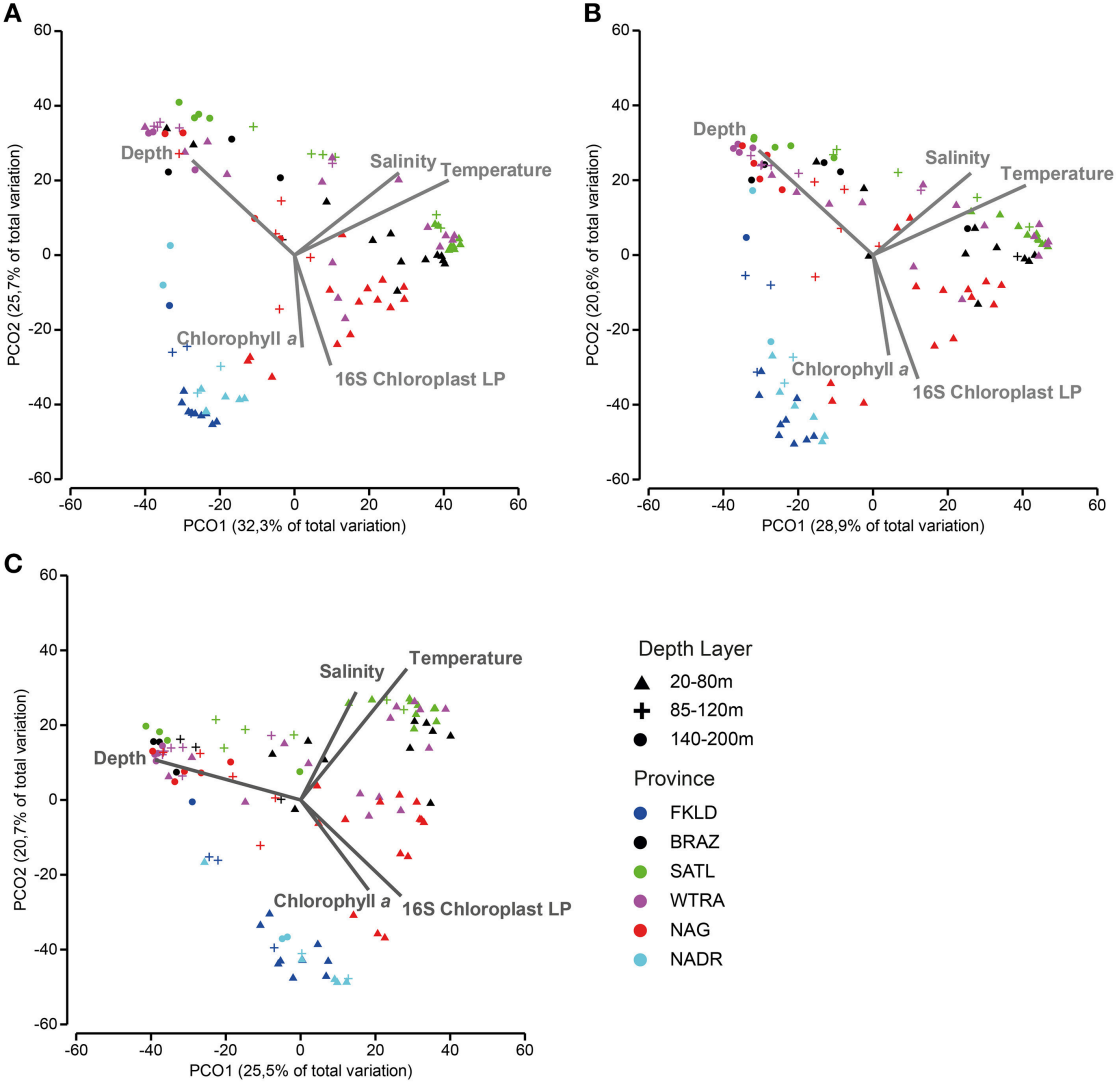
**Beta diversity patterns are shaped by environmental parameters and phytoplankton communities**. Principal coordinate analysis was used to show similarities among samples: **(A)** (FL), **(B)** (SPA), and **(C)** (LPA). In order to assess the effect of environmental parameters on the structure of the plot, Pearson correlation was calculated with the principal coordinates, after Bray-Curtis similarity was calculated on standardized abundance data at the OTU level. Only correlation higher than 0.4, with the first two axes were displayed on the chart area. The total amount of samples was reduced to match the biological data (bacterial abundances) with the environmental data (temperature, salinity, depth, and chlorophyll *a*) and with the relative abundance of phytoplankton (SP and LP). Color codes discriminate oceanographic provinces, while depth layers are shown with different symbols.

PCO of relative abundance of photosynthetic micro-eukaryotes showed a similar clustering of samples as that observed for bacteria (Figures 6A,B). The distance-decay relationship (Figures 6C,D) had a slightly weaker U-shape than that of bacteria (Figure S1). The marginal provinces FKLD and NADR grouped together, indicating similar photosynthetic micro-eukaryotic communities. A Mantel-like test (RELATE) showed strong significant (*p* < 0.002) correlations between photosynthetic eukaryote communities and bacterial communities from all size fractions of the plankton (Table 1). They were higher in the Southern hemisphere (R between 0.58 and 0.79) than in the Northern hemisphere (R between 0.30 and 0.42). The highest values were observed for FL with LP (*R* = 0.77) and LPA with LP (*R* = 0.79) in the Southern hemisphere, i.e., between micro-algae retrieved on the 8 μm filter and both the free living and large particle associated bacteria. By contrast, correlations between algae on the 3 μm filters and bacteria from either size class were relatively low, the lowest values being found for the correlation of SPA with SP (*R* = 0.30) and with LP (*R* = 0.33). Analysis of the chlorophyll *a* concentration and relative abundance of chloroplast 16S rRNA gene sequences showed that both were higher in the upper 80 m of the water column and in the two marginal provinces (FKLD and NADR) (Figure S5). Vector overlay of environmental parameters on the bacterial community composition showed that the two marginal provinces were discriminated from the other provinces of the transect by chlorophyll *a* concentration and the relative abundance of the 16S chloroplast sequences in the LP community rather than temperature, salinity and depth which instead were responsible for structuring the bathymetric stratification along the PCO1 (Figure 7).

**Table 1:** **Results of the RELATE test (Mantel-like test) between bacterioplankton communities photosynthetic micro-eukaryotes and chlorophyll *a* concentration based on Spearman rank correlation (999 permutations)**.

	**Part of the transect**	**Chloroplast 16S (SP)**	**Chloroplast 16S (LP)**	**Chlorophyll *a***
FL	South	0.59	0.77	0.3
	North	0.41	0.39	0.24
	Both	0.49	0.62	0.18
SPA	South	0.65	0.68	0.29
	North	0.3	0.33	0.44
	Both	0.32	0.51	0.26
LPA	South	0.58	0.79	0.32
	North	0.42	0.42	0.43
	Both	0.46	0.61	0.29

*Bray-Curtis similarity was calculated on standardized data for FL, SPA, LPA, SP, and LP communities. Chlorophyll a (μg/L) was normalized and Euclidean distance was calculated. All tests were statistically significant: P < 0.002*.

## Discussion

We investigated the biogeography of marine bacteria using ribosomal sequence data of bacterioplankton communities in the photic zone across ~12,000 km of the Atlantic Ocean. Based on deep sequencing of the V5-V6 region of the 16S rRNA gene from 382 filters it was possible to resolve bacterial community structure in three size classes per water sample. We found that the bacterial communities in these three size classes followed similar biogeographical patterns despite their different taxonomic compositions. This suggests that the driving forces of biogeography, e.g., dispersal, selection, mutation, and drift (Hanson et al., 2012) acted similarly on free-living and particle attached bacteria, in contrast to micro-eukaryotes whose biogeographical patterns depend on the size of the organism (Soininen, 2012).

### Distance decay-relationship in the Atlantic Ocean

Our analysis revealed a clear negative linear relationship between bacterial community similarity and distance for each hemisphere separately. However, when the complete transect was analyzed, after roughly 6000 km, community similarity increased again, reaching values between 40 and 60% Bray-Curtis similarity at the end of the transect. Our analysis suggests that the distance-decay relationship for marine bacterioplankton is dependent on the geographical scale that is analyzed, and there is no such relationship for the full transect. Our data are in accordance with the *Tara* Oceans study (Sunagawa et al., 2015) which reported a negative relationship between distance and community similarity within each oceanographic region (e.g., North Atlantic Ocean, South Atlantic Ocean) up to 5000 km. At larger distances the similarity increased again and no linear relationship was observed. However, differently from the work from Sunagawa and colleagues we investigated the community composition along a latitudinal transect sampled in 5 weeks, while the *Tara* oceans sampling sites aimed to cover as much as possible of the ocean with a sampling sheme that did not follow a latitudinal or longitudinal gradient. Furthermore, the huge amount of samples were collected in a time-frame of 4 years, and therefore also influenced by seasonal processes. It is therefore intriguing to notice that despite major differences in the sampling approach our results are in accordance with those from Sunagawa and colleagues, suggesting that the pattern is independently consistent irrespective of sampling design and time scale analyzed. A decrease in similarity of bacterial communities was not observed at distances beyond 5000 km in the global ocean. For archaea, a recent study focusing on AOA reported a “bipolar distribution” of AOA communities along a latitudinal transect in the Atlantic Ocean, with the marginal samples characterized by a higher similarity. This pattern was strongest in the epipelagic zone (Sintes et al., 2015). Thus, evidence is accumulating that the distance-decay concept from macro-ecology also exists in marine microbial ecology, but it is strongly context dependent (depth, geographical range, and perhaps season). The distance-decay relationship can be found for certain geographical scales (although interestingly not at distances larger than 6000 km), but local conditions (e.g., in the FKDL and NADR provinces) can select for highly similar communities across large distances, thus overriding the distance-decay concept entirely. Overall it seems that limits to dispersal do not appear to have a strong influence on the composition of marine microbial communities and rather selective processes shape bacterioplankton community composition.

It has been hypothesized that there is no real endemism in marine microbial communities and therefore most of marine diversity could be captured within 1 ml of seawater if it were only sequenced deep enough (Gibbons et al., 2013). Alternatively, microbes might be transported through the air. It was recently shown that millions of microbes per m^2^ leave and enter the ocean every day and can travel over distances of 11,000 km in roughly 4 days (Mayol et al., 2014).

### Longhurstian provincialism of bacterioplankton communities

The communities in the upper part of the photic zone were clearly dissimilar between provinces, particularly in the four intermediate provinces BRAZ, SATL, WTRA, and NAG. Different sets of OTUs were enriched in those oceanographic provinces, indicating that most of the OTUs were specialists with a narrow geographical distribution range rather than generalists. We can clearly demonstrate the province related distribution of bacterial taxa that had previously been suggested for the Eastern Atlantic Ocean but could not actually been shown with statistical support (Friedline et al., 2012). Provincialism in the Atlantic Ocean was recently also reported for AOA communities (Sintes et al., 2015). Studies carried out on the phylum Bacteroidetes in the North Atlantic also support the existence of distinct microbial communities in different oceanographic provinces (Gomez-Pereira et al., 2010, 2012). This is of particular interest since it indicates that oceanographic provinces harbor unique communities for all three domains of life and represent a powerful framework in microbial ecology.

### Vertical stratification of bacterioplankton communities

In contrast to the large variability of the communities in the upper photic zone, our analysis showed no distance decay in the deepest part of the photic zone for bacteria from all three size fractions of the plankton. Despite the huge distance covered, and the wide environmental gradient, the same OTUs were found across the complete transect in the 140–200 m depth layer. Most studies on stratification until now have been done on observatories at one spot, e.g., the Bermuda Atlantic Time-Series Station (BATS) (Treusch et al., 2009; Vergin et al., 2013) or compared extreme Arctic and Antarctic communities without any intermediate samples (Ghiglione et al., 2012).

### Environmental selection and specific influences on the Northern and Southern hemisphere

The relative contribution of geographical location to community composition was determined to be 7–9% of the total variation explained, in accordance with many studies as reviewed by Hanson et al. (2012) and suggesting a smaller role of dispersal limitation compared to environmental sorting (Hanson et al., 2012). When the two hemispheres were analyzed separately it increased to between 17 and 20% of the total variation in the Northern hemisphere, but remained at 4–5% in the Southern hemisphere. This suggests stronger dispersal processes in the Northern hemisphere. Along our transect we sampled the Mauritanian upwelling area where mixing processes occur (Longhurst, 1998). Moreover, in the Northern hemisphere we sampled along the border of the North Atlantic Gyre, while in the Southern hemisphere we crossed the South Atlantic Gyre (Longhurst, 1998). Overall it might be that our results reflect the hydrography of the area sampled. It is therefore possible that the Northen hemisphere is a more dynamic ecosystem characterized by extensive mixing processes that promote bacterial transportation across the basin (Agogue et al., 2011). In contrast it seems that the Southern hemisphere is mainly shaped by selective processes driven by temperature and depth and less extensive mixing processes take place.

Temperature was found to be the most important environmental driver for the whole transect (17–25%) as well in the Southern hemisphere (24–33%), while its effect was weaker in the Northern hermisphere, most strongly in the LPA community(3%). This might reflect the narrow temperature range found in the Northern hemisphere (12–27°C) compared to the southern one (3–28°C) (Milici et al., 2016b). For the *Tara* Oceans expedition temperature was shown to be the most important driver of community composition, explaining 61% of community diversity (Sunagawa et al., 2015). The difference to our data is most likely caused by the wider geographical and seasonal range analyzed in the *Tara* Oceans study, where it could also be that other parameters, not determined here, were more important in structuring microbial communities in the Atlantic Ocean at the time of sampling. Measurements of nutrient concentrations (phosphate and nitrate) along a latitudinal transect mirroring our sampling transect in the Atlantic Ocean have shown that nitrate concentration was higher in the proximity to the Mauritanian upwelling region and generally in the Northern hemisphere (Johnson et al., 2006) while phosphate and DOP (dissolved organic phosphate) were generally higher in the Southern hemisphere (Mather et al., 2008). Nutrients like nitrogen, phosphate, silicate and iron have a strong effect on bacterioplankton communities (Wietz et al., 2010; Bertrand et al., 2011; El-Swais et al., 2015).

### Influence of photosynthetic micro-eukaryotes on bacterial biogeography

The relatively high similarity of the microbial communities in the FKLD and NADR provinces is one of the main findings of our study. These provinces are characterized by high cell densities of photosynthetic micro-eukaryotes (Chust et al., 2013) and high chlorophyll *a* content throughout the year (De Monte et al., 2013). Analysis of the chloroplast 16S rRNA genes revealed that they contained similar photosynthetic eukaryotic taxa (Milici et al., 2016a). Taxonomic analysis of the bacterial communities (Milici et al., 2016a) showed an enrichment of taxa which are typically related to algae, e.g., Bacteroidetes, Gammaproteobacteria, and Rhodobacteriales (Teeling et al., 2012; Buchan et al., 2014; Wemheuer et al., 2014; Yang et al., 2015). It is tempting to speculate that photosynthetic micro-eukaryotes might have selected for specific microbial communities. This would be supported by studies on the seasonal succession of bacteria in the North Sea (Teeling et al., 2012) and elsewhere (Georges et al., 2014; El-Swais et al., 2015). Chemical analysis of the dissolved organic matter (DOM) produced by axenic phototrophic algae showed that its composition is partly taxon specific; closer related algae produce more similar DOM, thus they would be hypothesized to select for similar communities of heterotrophic bacteria (Becker et al., 2014). This hypothesis is also in accordance with a study investigating functional networks based on metatranscriptome data of a coastal and a pelagic marine site (Aylward et al., 2015), where the authors demonstrated that photosynthetic members of the community coordinated the transcriptional profile of heterotrophic bacteria. Furthermore, a recnt study (Needham and Fuhrman, 2016), has proposed that algal blooms are controlled by bacteria. In this study the authors suggested that interactions rather than physical parameters and inorganic nutrients determine the dominant species of an algal bloom. However, since correlations cannot imply causality, it is also possible that both photosynthetic micro-eukaryotes and bacterioplankton communities were shaped by unkown environmental factors, e.g., micro-nutrients like iron which can influence both communities (Falkowski et al., 1998; Bertrand et al., 2011), or phosphorous and nitrogen (Sylvan et al., 2006; Dyhrman et al., 2012; Zhao and Quigg, 2014; El-Swais et al., 2015).

## Conclusions

This work drew the following conclusions:

1. Bacterioplankton community similarity in the upper epipelagial of the Atlantic Ocean follows a latitudinal distance-decay relationship up to a distance of ~6000 km, afterwards community similarity increases again. Thus, dispersal limitation was relatively week and similar selective forces likely operated at both ends of the transect. The deepest analyzed layer of the photic zone (200 m) showed no significant distance-decay relationship across the entire transect.
2. Longhurstian provinces harbor distinct bacterioplankton communities, indicating that they represent a useful framework in microbial ecology.
3. The beta-diversity of photosynthetic micro-eukaryotes shows a similar pattern as that of bacteria and community composition of both is highly correlated. We hypothesize that interactions between micro-algae and bacteria might represent an important driver of bacterial community composition.

These analyses were performed for bacteria from three different size classes of the plankton. In spite of the substantial differences in the taxonomic composition of the respective microbial communities (Milici et al., 2016a), the biogeographical analyses yielded similar patterns, suggesting that the underlying mechanisms of dispersal and selection were similar. Altogether the data suggest that common macro-ecological concepts like the distance-decay relationship cannot be transferred to microbial communities, although the underlying mechanism like dispersal and selection hold for both microorganisms and macro-organisms but occur at different temporal and spatial scales.

## Author contributions

MM isolated the DNA, IP, DP, MW-O provided the method for sequencing and constructed the amplicon libraries. RJ performed bioinformatics analysis. MM, JT, and ZD analyzed the data. TB analyzed the oceanographic data, JD contributed to the data analysis. HG, MW, and MS measured environmental parameters. IW and HW collected the samples during the oceanographic cruise. MS organized the oceanographic cruise. IW supported and supervised the research. MM and IW wrote the manuscript, all authors reviewed the manuscript.

### Conflict of interest statement

The authors declare that the research was conducted in the absence of any commercial or financial relationships that could be construed as a potential conflict of interest.

## Supplementary material

Table S1 **Average values of temperature (°C) according to the oceanographic province of origin and depth layer**.

Table S2 **Average values of salinity (PSU) according to the oceanographic province of origin and depth layer**.

Table S3 **Sequencing results and metadata**. The sequencing results, after quality control, are reported for bacteria from each of the three size fractions of the plankton, FL, SPA and LPA. Sequences that were classified as chloroplast are collected separately and named: SP for those derived from the 3 μm filters and LP from those retrieved from the 8 μm filters. Geographical location, environmental parameters and factors used for multivariate statistic are listed for each sample.

Table S4 **PERMANOVA test was performed to assess the effect of province and depth layer in a crossed design (fixed effect of factors) on FL community**. Abundances of bacterial OTUs were used to calculate Bray-Curtis similarity after standardization. 999 permutations were performed with unrestricted permutation of raw data. Partial model was used for sum of squares. (DF) Degrees of freedom, (SS) Sum of squares, (MS) mean of squares.

Table S5 **PERMANOVA test was performed to assess the effect of province and depth layer in a crossed design (fixed effect of factors) on SPA community**. Abundances of bacterial OTUs were used to calculate Bray-Curtis similarity after standardization. 999 permutations were performed with unrestricted permutation of raw data. Partial model was used for sum of squares. (DF) Degrees of freedom, (SS) Sum of squares, (MS) mean of squares.

Table S6 **PERMANOVA test was performed to assess the effect of province and depth layer in a crossed design (fixed effect of factors) on LPA community**. Abundances of bacterial OTUs were used to calculate Bray-Curtis similarity after standardization. 999 permutations were performed with unrestricted permutation of raw data. Partial model was used for sum of squares. (DF) Degrees of freedom, (SS) Sum of squares, (MS) mean of squares.

Table S7 **Multivariate multiple linear regression (DISTLM) for FL community was performed with forward selection and adjusted *R*^2^ determination factor using environmental variables [temperature (°c), salinity (PSU), and depth (m)] and geographical location [latitude (°N) and longitude (°E)]**. Explanatory variables were normalized and Euclidean distance was calculated. Bacterial abundances were standardized and Bray-Curtis similarity was calculated.

Table S8 **Multivariate multiple linear regression (DISTLM) for SPA community was performed with forward selection and adjusted *R*^2^ determination factor using environmental variables [temperature (°c), salinity (PSU), and depth (m)] and geographical location [latitude (°N) and longitude (°E)]**. Explanatory variables were normalized and Euclidean distance was calculated. Bacterial abundances were standardized and Bray-Curtis similarity was calculated.

Table S9 **Multivariate multiple linear regression (DISTLM) for LPA community was performed with forward selection and adjusted *R*^2^ determination factor using environmental variables [temperature (°c), salinity (PSU), and depth (m)] and geographical location [latitude (°N) and longitude (°E)]**. Explanatory variables were normalized and Euclidean distance was calculated. Bacterial abundances were standardized and Bray-Curtis similarity was calculated.

Figure S1 **Distance decay relationship for the Atlantic Ocean bacterioiplankton**. Bray-Curtis similarity was calculated on standardized abundance data. Every dot represents a pairwise among samples belonging to the same size class; the similarity is plotted against geographical distance. Data are shown for the Southern and Northern hemisphere and the whole transect (labeled South, North, Both) from top to bottom. The three size fractions of the bacterioplankton are displayed from left to right. The color key indicates the distance between samples expressed as the number of oceanographic provinces. On the chart area of each graph the regression line model and second order polynomial curve which fitted the data are reported. All models were highly significant (*p* < 0.001).

Figure S2 **Distance decay relationship for small particle associated (SPA) communities along the water column**. Bray-Curtis similarity was calculated on standardized abundance data, and plotted against the geographical distance expressed in Km. All procedures and abbreviations are the same as in Figure 3.

Figure S3 **Distance decay relationship for large particle associated (LPA) communities along the water column**. Bray-Curtis similarity was calculated on standardized abundances data, and plotted against the geographical distance expressed in Km. All procedures and abbreviations are the same as in Figure 3.

Figure S4 **Province similarity along the water column for marginal provinces FKLD and NADR**. Bray-Curtis similarity was calculated on standardized abundance data. Depths were combined into two layers: 20–80 and 85–120 m. The average of the Bray-Curtis similarity was calculated for each of the depth layers of the province (FKLD and NADR, respectively) and compared against all six provinces. From top to down bacteria from the three size fractions of the plankton are shown. Color key shows different depth layers.

Figure S5 **Relative abundance of chloroplast 16S sequences and chlorophyll *a* concentration**. Samples are ordered along the x-axis from the Southern to the Northern edge of the transect. The five depth layers are shown separately 20 **(A)**, 40 **(B)**, 50–80 **(C)**, 85–120 **(D)**, and 140–200 m **(E)**. For each sample the relative abundance of 16S chloroplast reads, expressed in percentage, was calculated and plotted as a square. The color codes of the squares display the six oceanographic provinces. Chlorophyll *a* concentration, measured *in situ*, is plotted as a bar for each sample.
