# Supplementary material for: Bacterioplankton Biogeography of the Atlantic Ocean: A Case Study of the Distance-Decay Relationship
Source: Front Microbiol. 2016 Apr 26;7:590. doi: 10.3389/fmicb.2016.00590 (PMC4845060; doi:10.3389/fmicb.2016.00590)
Supplement: Table S1 — Average values of temperature (°C) according to the oceanographic province of origin and depth layer. [file TableS1-S2.docx]

Table S1: Average values of temperature (°C) according to the province of origin and depth layer.

|  |  |  |  | **Province** |  |  |  |
| --- | --- | --- | --- | --- | --- | --- | --- |
|  |  | **FKLD** | **BRAZ** | **SATL** | **WTRA** | **NAG** | **NADR** |
|  | **20** | 10.01 ± 1.73 | 21:42 ± 1.69 | 27:04 ± 0.64 | 25.43 ± 2.73 | 18.71 ± 1.65 | 13.16 ± 0.82 |
| **Depth layer** | **40** | 9.64 ± 1.45 | 21:60 ± 1.05 | 26:68 ± 1.63 | 22.49 ± 3.84 | 18.33 ± 1.67 | 13.08 ± 0.85 |
| **(m)** | **50-80** | 8.73 ± 1 | 18:94 ± 0.81 | 26:6 ± 1.18 | 20.84 ± 3 | 18.52 ± 1.7 | 12.42 ± 0.6 |
|  | **85-120** | 6.26 ± 1.77 | 17:25 ± 0.51 | 23:71 ± 1.88 | 16.41 ± 1.67 | 17.49 ± 1.5 | 12.43 ± 0.61 |
|  | **140-200** | 4.54 | 15:46 ± 0.8 | 19:07 ± 4.11 | 12.66 ± 6.13 | 15.97 ± 1.93 | 12.04 ± 0.33 |

Table S2: Average values of salinity (PSU) according to the province of origin and depth layer.

|  |  |  |  | **Province** |  |  |  |
| --- | --- | --- | --- | --- | --- | --- | --- |
|  |  | **FKLD** | **BRAZ** | **SATL** | **WTRA** | **NAG** | **NADR** |
|  | **20** | 33.52 ± 0.53 | 35:64 ± 0.28 | 37:01 ± 0.38 | 35.95 ± 0.17 | 36.64 ± 0.2 | 35.72 ± 0.1 |
| **Depth layer** | **40** | 33.53 ± 0.51 | 35:76 ± 0.08 | 37:11 ± 0.28 | 36.04 ± 0.25 | 36.61 ± 0.16 | 35.72 ± 0.1 |
| **(m)** | **50-80** | 33.52 ± 0.50 | 35:85 ± 0.09 | 37:05 ± 0.23 | 35.96 ± 0.31 | 36.67 ± 0.17 | 35.72 ± 0.06 |
|  | **85-120** | 33.54 ± 0.50 | 35:78 ± 0.13 | 37:06 ± 0.25 | 35.69 ± 0.23 | 36.57 ± 0.2 | 35.74 ± 0.09 |
|  | **140-200** | 34.05 | 35:64 ± 0.12 | 36:22 ± 0.64 | 35.30 ± 0.31 | 36.27 ± 0.29 | 35.68 ± 0.04 |
