# Supplementary material for: Bacterioplankton Biogeography of the Atlantic Ocean: A Case Study of the Distance-Decay Relationship
Source: Front Microbiol. 2016 Apr 26;7:590. doi: 10.3389/fmicb.2016.00590 (PMC4845060; doi:10.3389/fmicb.2016.00590)
Supplement: Table S4 — PERMANOVA test was performed to assess the effect of province and depth layer in a crossed design (fixed effect of factors) on FL community. Abundances of bacterial OTUs were used to calculate Bray-Curtis similarity after standardization. 999 permutations were performed with unrestricted permutation of raw data. Partial model was used for sum of squares. (DF) Degrees of freedom, (SS) Sum of squares, (MS) mean of squares. [file TableS4-S9.docx]

Table S4: PERMANOVA test was performed to assess the effect of province and depth layer in a crossed design (fixed effect of factors) on FL community. Abundances of bacterial OTUs were used to calculate Bray-Curtis similarity after standardization. 999 permutations were performed with unrestricted permutation of raw data. Partial model was used for sum of squares. (DF) Degrees of freedom, (SS) Sum of squares, (MS) mean of squares.

| **Source** | **DF** | **SS** | **MS** | **Pseudo-F** | **R^2^** | **P value** |
| --- | --- | --- | --- | --- | --- | --- |
| **Province**  **Depth layer**  **Depth layer X Province**  **Residual**  **Total** | 5  4  20  100  129 | 104200  48046  41415  84323  319120 | 20840  12012  2070.8  843.23 | 24.71  14.24  2.46 | 31.66  23.44  17.27 | 0.001  0.001  0.001 |

Table S5: PERMANOVA test was performed to assess the effect of province and depth layer in a crossed design (fixed effect of factors) on SPA community. Abundances of bacterial OTUs were used to calculate Bray-Curtis similarity after standardization. 999 permutations were performed with unrestricted permutation of raw data. Partial model was used for sum of squares. (DF) Degrees of freedom, (SS) Sum of squares, (MS) mean of squares.

| **Source** | **DF** | **SS** | **MS** | **Pseudo-F** | **R^2^** | **P value** |
| --- | --- | --- | --- | --- | --- | --- |
| **Province**  **Depth layer**  **Depth layer X Province**  **Residual**  **Total** | 5  4  20  95  124 | 98832  46044  43105  120290  343750 | 19766  11511  2155.3  1266.2 | 15.61  9.09  1.70 | 30.87  22.62  14.93 | 0.001  0.001  0.001 |

Table S6: PERMANOVA test was performed to assess the effect of province and depth layer in a crossed design (fixed effect of factors) on LPA community. Abundances of bacterial OTUs were used to calculate Bray-Curtis similarity after standardization. 999 permutations were performed with unrestricted permutation of raw data. Partial model was used for sum of squares. (DF) Degrees of freedom, (SS) Sum of squares, (MS) mean of squares.

| **Source** | **DF** | **SS** | **MS** | **Pseudo-F** | **R^2^** | **P value** |
| --- | --- | --- | --- | --- | --- | --- |
| **Province**  **Depth layer**  **Depth layer X Province**  **Residual**  **Total** | 5  4  20  101  130 | 93496  47942  45049  115940  334160 | 18699  1985  2252.5  1147.9 | 16.29  10.41  1.96 | 29.5  23.04  16.29 | 0.001  0.001  0.001 |

Table S7: Multivariate multiple linear regression (DISTLM) was performed with forward selection and adjusted R^2^ determination factor using environmental variables (temperature (°c), salinity (PSU) and depth (m)) and geographical location (latitude (°N) and longitude (°E)). Explanatory variables were normalized and Euclidean distance was calculated. Bacterial abundances were standardized and Bray-Curtis similarity was calculated.

| **Bacterial community** | **Variable** | **SS** | **Pseudo-F** | **P value** | **Adjusted R^2^** | **Cumulative %** |
| --- | --- | --- | --- | --- | --- | --- |
| **South Hemisphere FL**  **North hemisphere**  **FL**  **All transect**  **FL** | Temperature (°C)  Depth (m)  Latitude (°N)  Longitude (°E)  Salinity (PSU)  Temperature (°C)  Latitude (°N)  Depth (m)  Longitude (°E)  Salinity (PSU  Temperature (°C)  Depth (m)  Latitude (°N)  Longitude (°E)  Salinity (PSU) | 61222  41176  6805.9  3636.2  2821.4  26605  20512  8325.6  5424.8  5473.2  80902  53093  14987  10782  6075.4 | 36.62  37.45  6.97  3.41  2.97  20.84  11.81  7.28  5.57  5.16  43.7  36.76  11.22  8.56  4.98 | 0.001  0.001  0.001  0.01  0.017  0.001  0.001  0.001  0.001  0.001  0.001  0.001  0.001  0.001  0.001 | 0.334  0.228  0.034  0.015  0.011  0.222  0.162  0.064  0,041  0.040  0.250  0.165  0.044  0.031  0.016 | 33.4  56.2  59.6  61.1  62.2  22.2  38.4  44.8  48.9  52.8  25.0  41.5  45.9  49.0  50.5 |

Table S8: Multivariate multiple linear regression (DISTLM) was performed with forward selection and adjusted R^2^ determination factor using environmental variables (temperature (°c), salinity (PSU) and depth (m)) and geographical location (latitude (°N) and longitude (°E)). Explanatory variables were normalized and Euclidean distance was calculated. Bacterial abundances were standardized and Bray-Curtis similarity was calculated.

| **Bacterial community** | **Variable** | **SS** | **Pseudo-F** | **P value** | **Adjusted R^2^** | **Cumulative %** |
| --- | --- | --- | --- | --- | --- | --- |
| **South Hemisphere**  **SPA**  **North hemisphere**  **SPA**  **All transect**  SPA | Temperature (°C)  Depth (m)  Longitude (°E)  Salinity (PSU)  Latitude (°N)  Temperature (°C)  Latitude (°N)  Depth (m)  Salinity (PSU  Longitude (°E)  Temperature (°C)  Depth (m)  Latitude (°N)  Longitude (°E)  Salinity (PSU) | 56640  33140  6298.54322.5  3965.8  30369  20336  13449  5122.34647.8  79232  50851  19139  9573.1  6629.2 | 30.70  23.94  5.15  3.22  3.09  15.361  12.576  9.74  3.93  3.77  36.84  29.03  11.9  6.21  4.42 | 0.001  0.001  0.001  0.005  0.005  0.001  0.001  0.001  0.001  0.001  0.001  0.001  0.001  0.001  0.001 | 0.298  0.175  0.029  0.017  0.015  0.213  0.143  0.094  0.030  0.028  0.224  0.144  0.052  0.024  0.015 | 29.8  47.3  50,2  51.9  53.4  21.3  35.6  45  48  50.8  22.4  36.8  42.0  44.4  45.9 |

Table S9: Multivariate multiple linear regression (DISTLM) was performed with forward selection and adjusted R^2^ determination factor using environmental variables (temperature (°c), salinity (PSU) and depth (m)) and geographical location (latitude (°N) and longitude (°E)). Explanatory variables were normalized and Euclidean distance was calculated. Bacterial abundances were standardized and Bray-Curtis similarity was calculated.

| **Bacterial community** | **Variable** | **SS** | **Pseudo-F** | **P value** | **Adjusted R^2^** | **Cumulative %** |
| --- | --- | --- | --- | --- | --- | --- |
| **South Hemisphere**  **LPA**  **North hemisphere**  **LPA**  **All transect**  LPA | Temperature (°C)  Depth (m)  Latitude (°N)  Longitude (°E)  Salinity (PSU)  Depth (m)  Latitude (°N)  Salinity (PSU  Longitude (°E)  Temperature (°C)  Temperature (°C)  Depth (m)  Latitude (°N)  Longitude (°E)  Salinity (PSU) | 45403  31004  6568.7  4992  3866.6  30185  20476  10209  5064.8  4969  60401  52292  21616  9749.1  6291.5 | 25.95  21.04  4.88  3.51  2.96  17.13  14.34  9.91  4.36  4.56  28.46  30.22  13.73  6.46  4.28 | 0.001  0.001  0.001  0.002  0.006  0.001  0.001  0.001  0.001  0.001  0.001  0.001  0.001  0.001  0.001 | 0.244  0.168  0.031  0.021  0.015  0.218  0.148  0.087  0.032  0.032  0.174  0.152  0.061  0.025  0.015 | 24.4  41.2  44.3  46.4  47.9  21.8  36.6  45.3  48.5  51.6  17.4  32.7  38.8  41.3  42.8 |
