# Supplementary figures and images for: Bacterioplankton Biogeography of the Atlantic Ocean: A Case Study of the Distance-Decay Relationship
Source: Front Microbiol. 2016 Apr 26;7:590. doi: 10.3389/fmicb.2016.00590 (PMC4845060; doi:10.3389/fmicb.2016.00590)

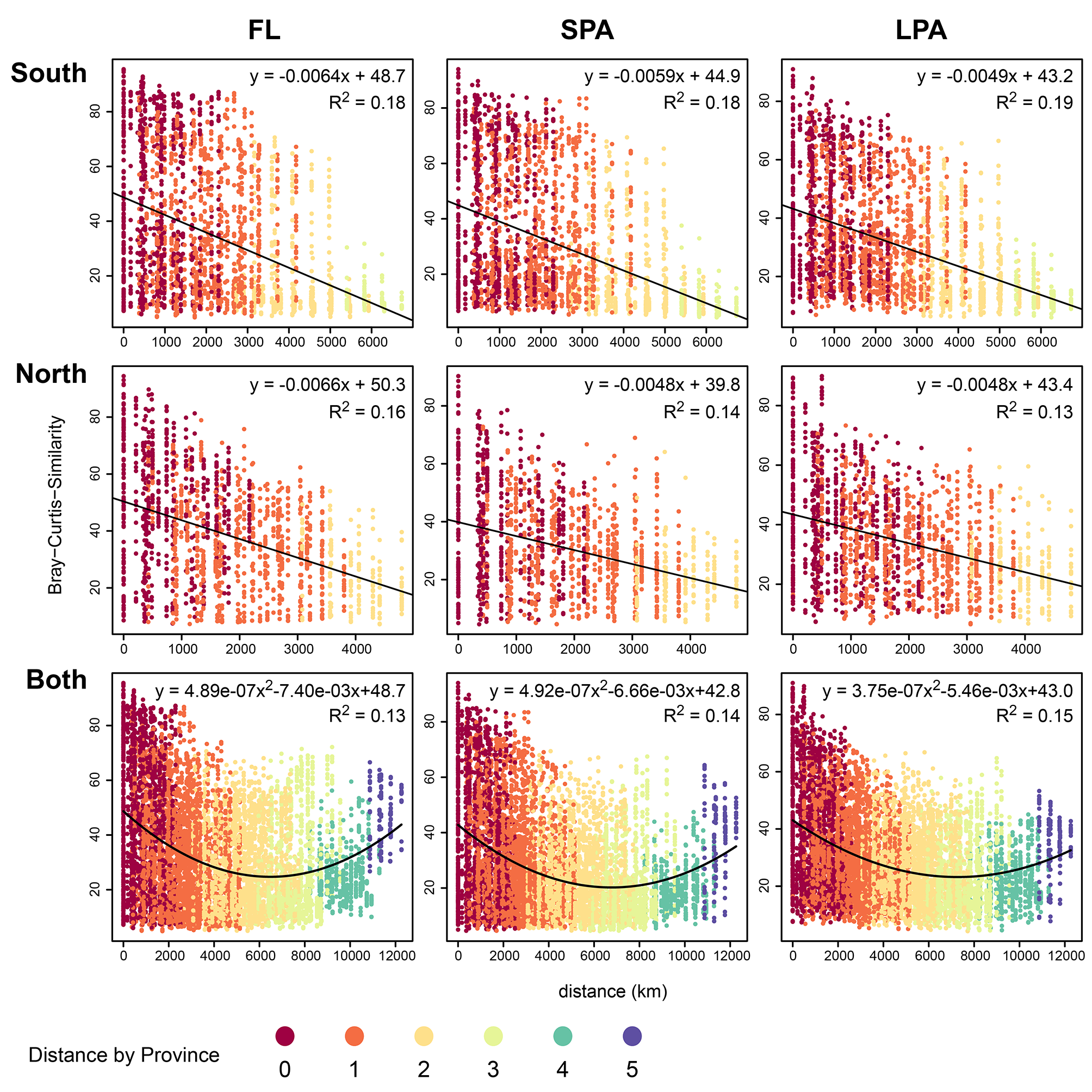

Supplement: Figure S1 — Distance decay relationship for the Atlantic Ocean bacterioiplankton. Bray-Curtis similarity was calculated on standardized abundance data. Every dot represents a pairwise among samples belonging to the same size class; the similarity is plotted against geographical distance. Data are shown for the Southern and Northern hemisphere and the whole transect (labeled South, North, Both) from top to bottom. The three size fractions of the bacterioplankton are displayed from left to right. The color key indicates the distance between samples expressed as the number of oceanographic provinces. On the chart area of each graph the regression line model and second order polynomial curve which fitted the data are reported. All models were highly significant (p < 0.001). [file Image1.TIF]

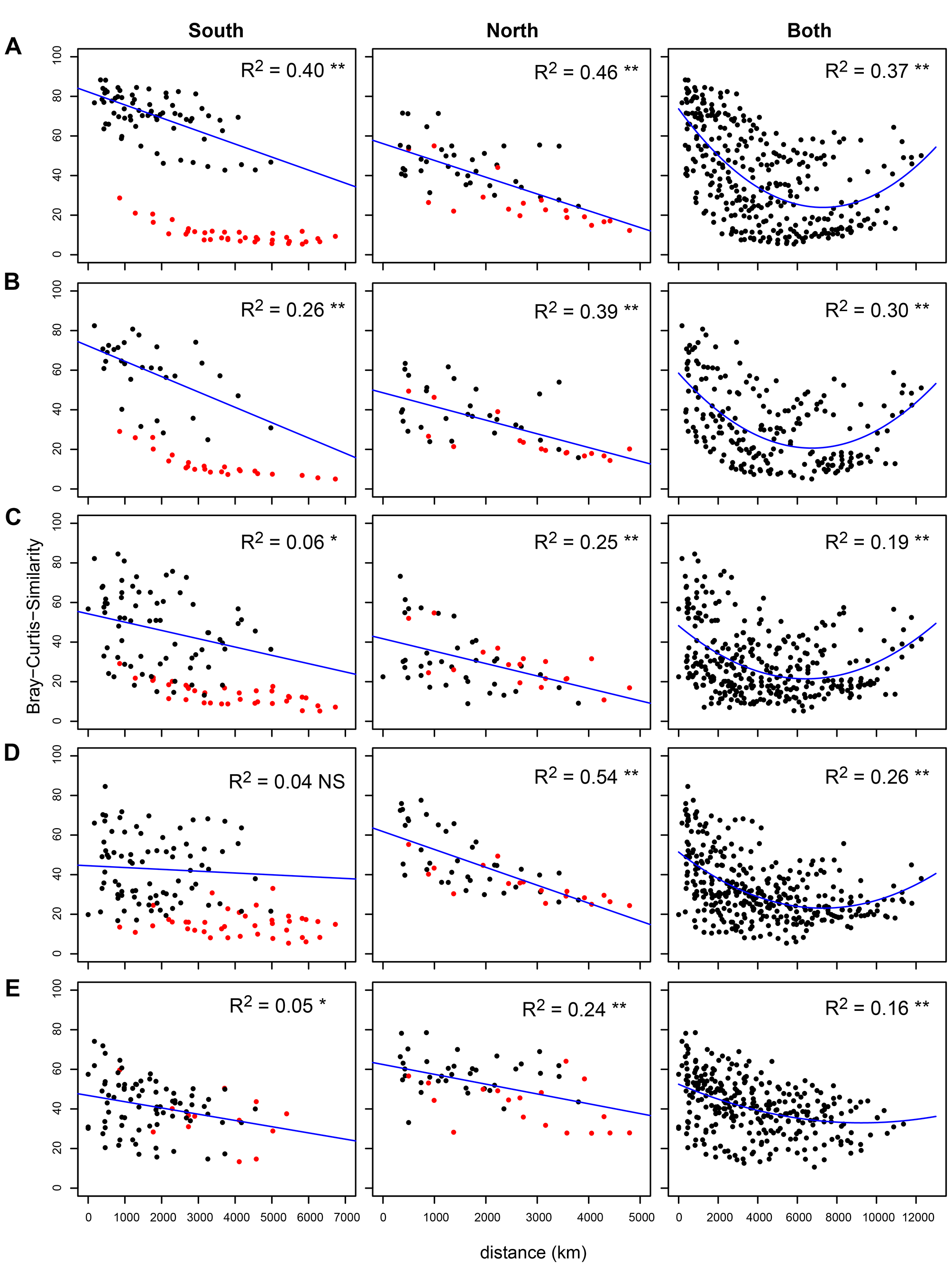

Supplement: Figure S2 — Distance decay relationship for small particle associated (SPA) communities along the water column. Bray-Curtis similarity was calculated on standardized abundance data, and plotted against the geographical distance expressed in Km. All procedures and abbreviations are the same as in Figure 3. [file Image2.TIF]

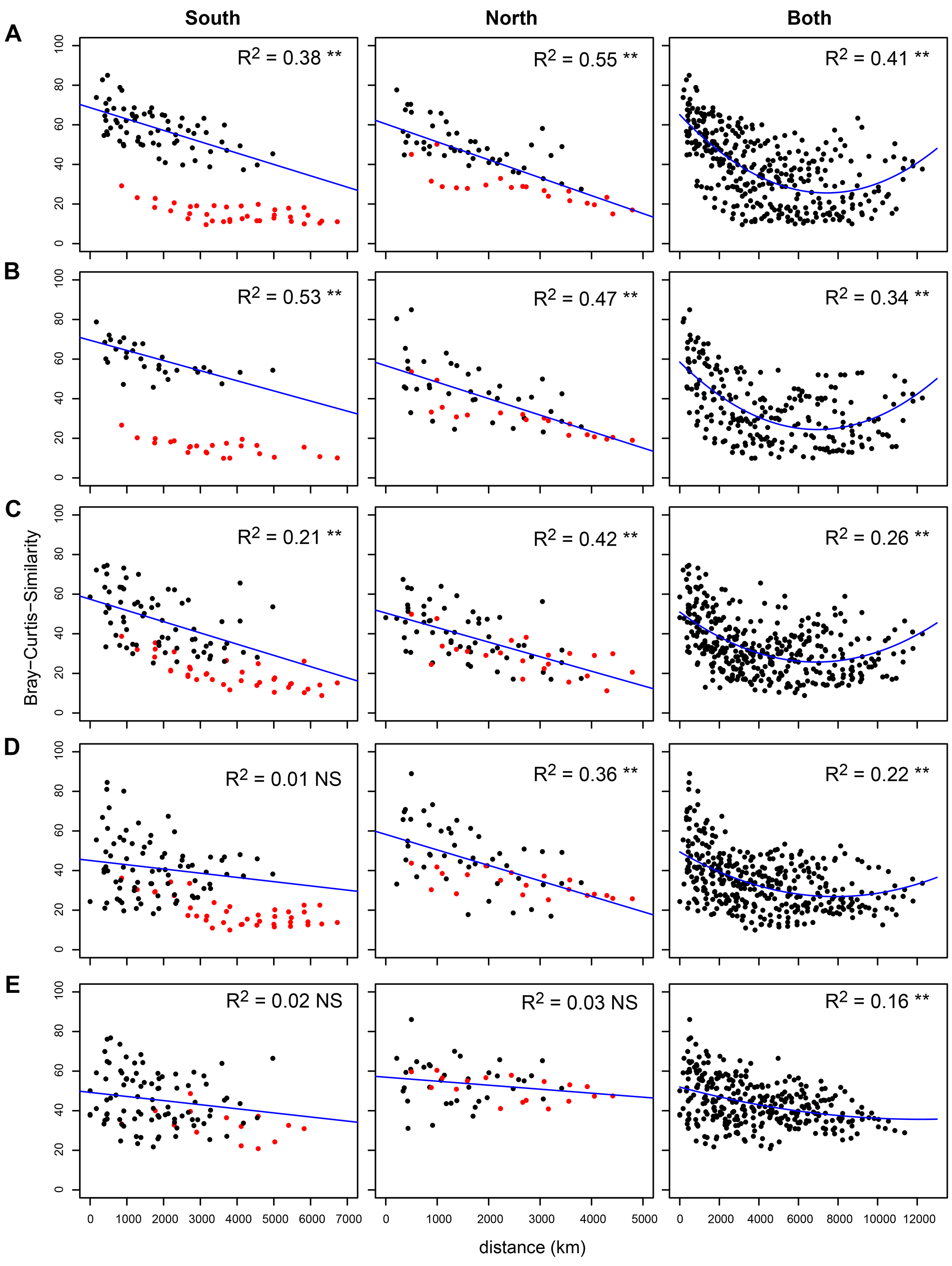

Supplement: Figure S3 — Distance decay relationship for large particle associated (LPA) communities along the water column. Bray-Curtis similarity was calculated on standardized abundances data, and plotted against the geographical distance expressed in Km. All procedures and abbreviations are the same as in Figure 3. [file Image3.TIF]

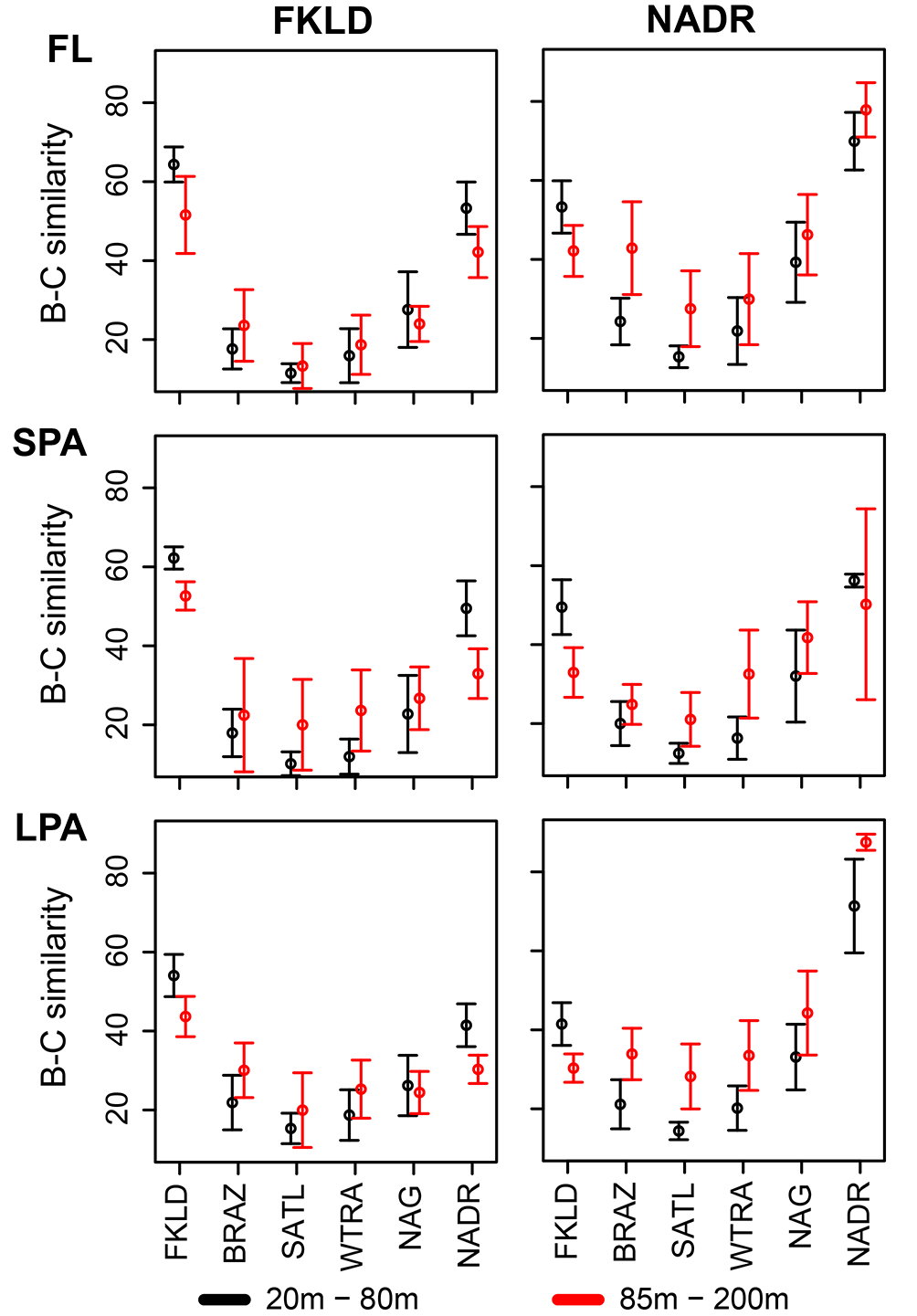

Supplement: Figure S4 — Province similarity along the water column for marginal provinces FKLD and NADR. Bray-Curtis similarity was calculated on standardized abundance data. Depths were combined into two layers: 20–80 and 85–120 m. The average of the Bray-Curtis similarity was calculated for each of the depth layers of the province (FKLD and NADR, respectively) and compared against all six provinces. From top to down bacteria from the three size fractions of the plankton are shown. Color key shows different depth layers. [file Image4.TIF]

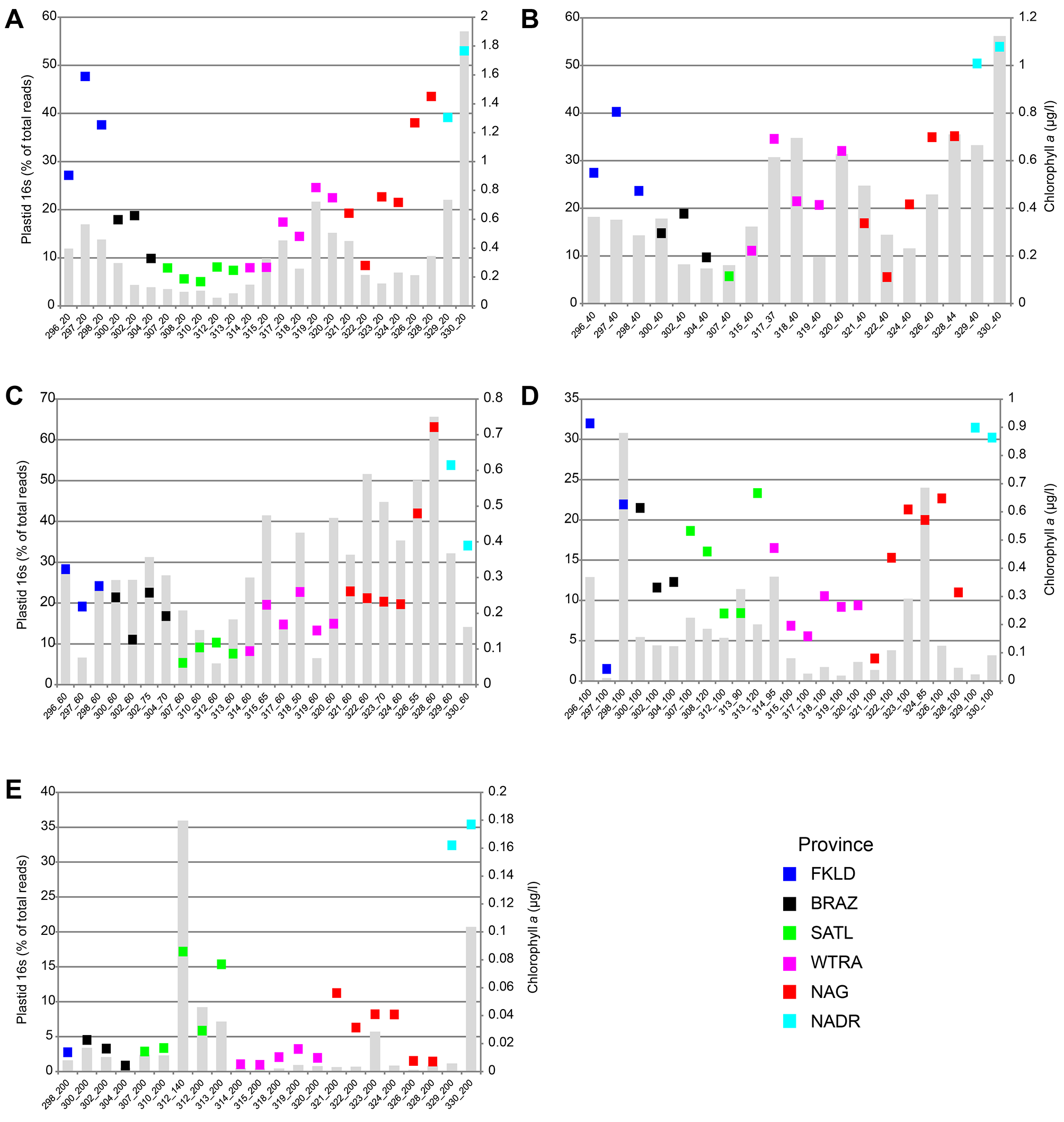

Supplement: Figure S5 — Relative abundance of chloroplast 16S sequences and chlorophyll a concentration. Samples are ordered along the x-axis from the Southern to the Northern edge of the transect. The five depth layers are shown separately 20 (A), 40 (B), 50–80 (C), 85–120 (D), and 140–200 m (E). For each sample the relative abundance of 16S chloroplast reads, expressed in percentage, was calculated and plotted as a square. The color codes of the squares display the six oceanographic provinces. Chlorophyll a concentration, measured in situ, is plotted as a bar for each sample. [file Image5.TIF]
